# Layer-by-Layer Integration of Electrospun Nanofibers in FDM 3D Printing for Hierarchical Composite Fabrication

**DOI:** 10.3390/polym18010078

**Published:** 2025-12-27

**Authors:** Jaymin Vrajlal Sanchaniya, Hilary Smogor, Valters Gobins, Vincent Noël, Inga Lasenko, Simas Rackauskas

**Affiliations:** 1 Institute of Mechanical and Biomedical Engineering, Faculty of Civil and Mechanical Engineering, Riga Technical University, 6B Kipsala Street, LV-1048 Riga, Latvia; inga.lasenko@rtu.lv; 2 NETZSCH Instrumenty, Halicka 9, 31-036 Krakow, Poland; 3 Laboratory of Environmental Genetics, Institute of Biology, Faculty of Biology, Latvian University, Jelgavas Street 1, LV-1004 Riga, Latvia; 4 Université Paris Cité, ITODYS, CNRS, UMR 7086, 15 rue Jean Antoine de Baif, 75013 Paris, France; vincent.noel@u-paris.fr; 5 Institute of Materials Science, Kaunas University of Technology, K. Barsausko St. 59, 51423 Kaunas, Lithuania

**Keywords:** electrospinning, additive manufacturing, fused deposition modeling (FDM), composite, hierarchical structures

## Abstract

This study presents a novel integrated manufacturing approach that combines fused deposition modeling (FDM) 3D printing with in situ electrospinning to fabricate hierarchical composite structures composed of polylactic acid (PLA) reinforced with polyacrylonitrile (PAN) nanofibers. A mounting fixture was employed to enable layer-by-layer nanofiber deposition directly onto printed PLA layers in a continuous automated process, eliminating the need for prefabricated electrospun nanofiber mats. The influences of nozzle temperature (210–230 °C) and electrospinning time (5–15 min per layer) on mechanical, thermal, and morphological properties were systematically investigated. Optimal performance was achieved at an FDM nozzle temperature of 220 °C with 5 min of electrospinning time (sample E1), showing a 36.5% increase in tensile strength (71 MPa), a 33.3% increase in Young’s modulus (2.8 GPa), and a 62.0% increase in flexural strength (128 MPa) compared with the neat PLA. This enhancement resulted from the complete infiltration of molten PLA into the thin nanofiber mats, creating true fiber–matrix integration. Excessive nanofiber content (15 min ES) caused a 36.5% reduction in strength due to delamination and incomplete infiltration. Thermal analysis revealed a decrease in glass transition temperature (1.2 °C) and onset of thermal degradation (5.3–15.2 °C) with nanofiber integration. Fracture morphology confirmed that to achieve optimal properties, it was critical to balance the nanofiber reinforcement content with the depth of infiltration, as excessive content created poorly bonded interleaved layers. This integrated fabrication platform enables the production of lightweight hierarchical composites with multiscale, custom-made reinforcement for applications in biomedical scaffolds, protective equipment, and structural components.

## 1. Introduction

The development of fused deposition modeling (FDM) technology in additive manufacturing has revolutionized manufacturing by enabling the creation of complex objects at the microscale [1,2,3,4]. However, FDM-printed structures often exhibit mechanical limitations in strength and layer adhesion [5,6,7]. Similarly, the electrospinning technique generates nanoscale fibers through electrostatic forces from polymer solutions. An electrically charged jet produces ultrafine fibers when high voltage is applied during the process, while the solvent evaporates [8,9]. The fibers produced through electrospinning exhibit multiple advantages, including large surface area and porosity, together with mechanical flexibility and the ability to embed functional materials for applications in tissue engineering [10,11,12,13], filtration systems [14,15,16], sensors [17,18,19,20], drug delivery systems [21,22,23,24], and composite reinforcement [25,26,27].

A combined system that integrates FDM and electrospinning technology enables us to overcome the individual limitations of these technologies through optimized performance benefits [28,29,30]. This combined approach addresses critical industrial challenges by creating solid structures with improved mechanical and thermal performance, improved surface properties, and multifunctional capabilities [31,32]. These hybrid materials possess important material properties, such as anisotropy, high strength-to-weight ratios, and controlled degradation profiles that are valuable for advanced engineering applications.

Recent studies have explored different techniques for embedding nanofibers into 3D-printed structures to improve their mechanical and functional properties. He and Molnár [33] developed a technique to interleave electrospun polylactic acid (PLA) nanofiber mats between printed PLA layers during the FDM process. Their results showed that the integration of nanofiber interleaves led to significant improvements in mechanical properties, with increases in storage modulus (10.3%), tensile strength (16.5%), and Young’s modulus (34.3%) compared to the neat PLA specimens. Kara et al. [34] introduced a novel 3D printing method that combines material extrusion and melt blending to create hierarchical structures containing both nano/microfibers and solid or infill layers. The resulting structures showed improved mechanical properties, with fiber layers that enhanced the crystallinity of the printed products and provided reinforcement. Moukbil et al. [35] analyzed the effect of the concentration of bovine hydroxyapatite (BHA) on tricalcium phosphate (TCP) and polycaprolactone (PCL) composite scaffolds fabricated via 3D printing. Their results showed that bioactivity increased systematically, while cell proliferation rates and growth rates improved, as the BHA concentration reached 15% (wt./wt.) in 3D-printed materials, showing the importance of material composition to achieve functional 3D-printed structures.

Many researchers in the field of bone tissue engineering have studied the integration of nanofibers within 3D-printed scaffolds. Khodabandeh et al. [36] produced microfibrous composite scaffolds made of polycaprolactone (PCL)/hydroxyapatite (HA) using near-field electrospinning (NFES) and subsequently integrated aligned and random electrospun poly(L-lactic acid) (PLLA) nanofibers to improve mechanical properties and cell adhesion. With directional nanofiber arrangement, the biocomponents showed substantial developmental improvements in terms of alkaline phosphatase action, together with improved mineral development and structural characteristics via enhancement of the elastic modulus by 268% and the ultimate tensile strength by 130%, compared to microfibrous scaffolds. Similarly, Belgheisi et al. [37] developed a PCL/HA microfibrous composite scaffold prepared by NFES with a fiber spacing of 500 μm. Their findings showed that electrospun PLLA nanofibers, once added to the microfibrous scaffold, increased cell adhesion by 334% compared to the microfibrous scaffold without nanofiber integration, and the aligned nanofibers exhibited piezoelectric properties that enhanced bone healing. Lackner et al. [38] produced anisotropic nanofiber composites via 3D printing of nanocellulose/alginate/CaCO_3_ inks with different fiber orientations. By controlling the orientation of the fiber printing path, they achieved flexibility and tensile modulus ranging from 1 to 30 MPa in the wet state.

Despite advances in combining 3D printing and electrospinning technologies, current approaches have significant operational restrictions. Most current approaches follow sequential methods, where pre-fabricated electrospun mats are manually inserted between printed layers during pauses in the printing process [33]. These sequential process methods lead to poor integration between the fibers and the matrix, nonuniform distribution of nanofibers, and procedural inefficiencies. However, the proposed approach integrates electrospinning directly into the 3D printing cycle through a mounting fixture, which allows for continuous layer-by-layer nanofiber deposition without manual intervention or process interruption. This integrated operation would address these limitations and potentially lead to more homogeneous and mechanically improved composite structures.

In this research, the specific combination of polylactic acid (PLA) and polyacrylonitrile (PAN) was strategically chosen for this hybrid fabrication approach to maximize both processability and performance benefits. PLA is widely used in FDM 3D printing due to its printability, relatively low melting temperature range (150–160 °C) [39], biodegradability [40], and biocompatibility [41]. These properties make it ideal for creating the structural framework of the composite. PAN was selected for the electrospun nanofiber component due to its outstanding mechanical properties, with tensile strength values typically 5–10 times higher than those of PLA nanofibers, excellent thermal stability, and a glass transition temperature of approximately 85–95 °C [42,43,44] compared to 55–65 °C for PLA [45,46,47]. The combination of PLA and PAN nanofibers in a single fabrication process produces a composite material with enhanced structural strength, improved ease of processing, and robust mechanical reinforcement and thermal properties, which have not been achieved with previous fabrication methods.

The hierarchical composite structures produced through this integrated approach have potential for diverse engineering applications. The combination of enhanced mechanical properties, controlled deposition of nanofibers, and tailored degradation profiles makes these materials particularly suitable for biomedical scaffolds, lightweight structural components, filtration membranes, and protective equipment where multi-scale reinforcement is advantageous.

This study presents a novel approach for producing 3D-printed solid structures along with electrospinning nanofibers using a 3D-printed mounting bracket, integrated system. The system operates in a configuration in which the electrospinning nozzle operates in sequence with the FDM extruder, allowing for precise deposition of nanofibers between printed layers. This approach offers several advantages: (1) improved mechanical properties through the incorporation of high-strength PAN nanofibers throughout the solid structure; (2) possible changes in crystallization patterns and thermal degradation patterns that can be attributed to the addition of polyacrylonitrile (PAN) nanofibers; and (3) reduced production time by eliminating separate processing steps. A thorough investigation of the morphological, mechanical, and thermal properties of these new composite structures will help us understand their structure–function relationships while suggesting potential applications of this integrated processing technique.

## 2. Materials and Methods

### 2.1. Materials

To fabricate hierarchical composite structures through an approach that integrates 3D FDM printing with electrospun nanofibers, a polymeric filament for 3D printing and the primary polymer material, along with the appropriate solvent, were utilized for the electrospinning process.

Commercial-grade polylactic acid (PLA) filament (Prusament PLA; 1.75 mm in diameter, density: 1.24 g/cm^3^) was purchased from Prusa Research a.s. (Partyzánská 188/7a, 17000 Prague, Czech Republic) and used as the base material for FDM 3D printing.

For the electrospinning process, polyacrylonitrile (PAN) with an average molecular weight (MW) of 150,000 (typical) and a CAS number of 25014-41-9 was obtained from Sigma-Aldrich Chemicals (Merck KGaA, Steinheim, Germany). N,N-Dimethylformamide (DMF), used as the solvent to prepare the electrospinning solution, was of ACS reagent grade with 99.8% purity (CAS number: 68-12-2) and was also purchased from Sigma-Aldrich Chemicals (Merck KGaA, Steinheim, Germany).

### 2.2. Fabrication of Hierarchical Structures

**Figure 1:**
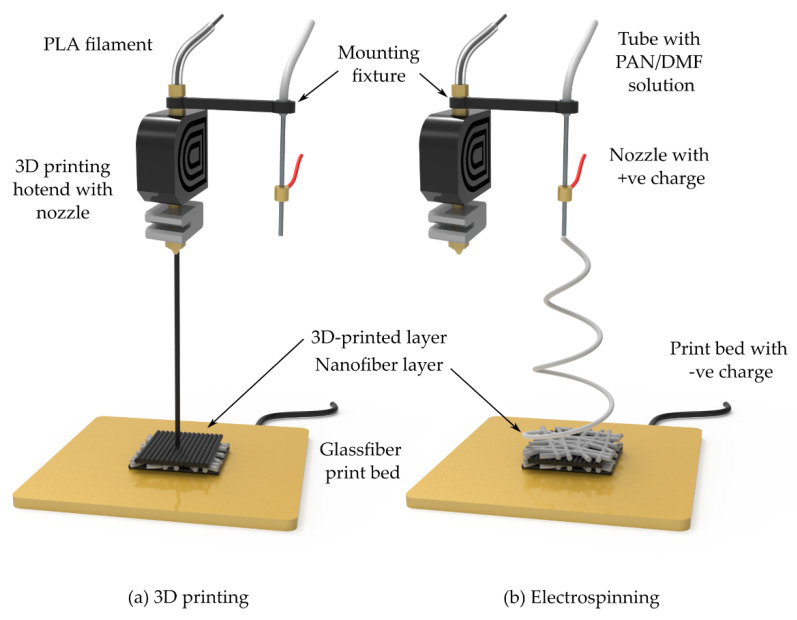
Schematic illustration of the hybrid setup for fabricating hierarchical structures: (**a**) FDM 3D printing process showing the PLA filament being extruded through the hotend nozzle onto the glass fiber print bed, and (**b**) electrospinning process showing the PAN/DMF solution being delivered through the needle with positive charge, producing nanofibers that are deposited onto the negatively charged print bed with the previously printed 3D layer.

The PAN nanofibers were synthesized using an electrospinning setup, following the protocol previously described by the authors [8,9,48]. To prepare the PAN solution, 10% (wt./wt.) PAN powder was thoroughly dissolved in N,N-dimethylformamide (DMF) under continuous stirring at 80 °C and 1000 rpm for 4 h using a magnetic stirrer. The solution was then electrospun using a 50 mL Luer-lock syringe equipped with an 18-gauge needle (outer diameter: 1.27 ± 0.01 mm; inner diameter: 0.838 ± 0.01 mm). The syringe was connected to the needle via a PTFE tube (outer diameter: 6.0 ± 0.01 mm; inner diameter: 4.0 ± 0.01 mm), and the needle was mounted to the print head using a 3D-printed mounting fixture, as illustrated in Figure 1. The electrospinning process was carried out under the following conditions: an applied voltage of 18 kV, a solution feed rate of 0.8 mL/h, and a working distance of 15 cm between the tip of the needle and the print bed.

For 3D printing, PLA filament was pre-dried in an eSUN eBOX Lite 3D Filament Dryer Box (Shenzhen Esun Industrial Co., Ltd., Shenzhen, China) for 24 h at 45 °C to remove moisture, following standard protocols for PLA filament preparation. All 3D printing and electrospinning parameters, as detailed in Table 1, were kept constant across experiments.

The integrated fabrication process involved automated sequential, layer-by-layer deposition of both printed and electrospun materials controlled through G-code programming. After each PLA layer, the tool head was automatically moved to a designated parking position where the needle was positioned 15 cm away from the print bed. Then, electrospinning was performed for 10 min without manual intervention, depositing PAN nanofibers directly onto the printed layer. Following nanofiber deposition, the next PLA layer was automatically printed, and this cycle was repeated throughout the build process. For the neat PLA specimens (without nanofibers), the tool head remained in the parking position for 10 min after each layer to allow the printed layer to solidify and cool, ensuring comparable thermal histories between samples with and without nanofibers. All experiments were carried out under controlled environmental conditions at 23 ± 1 °C and 40 ± 5% relative humidity. The experimental parameters investigated in this study are summarized in Table 2.

To systematically evaluate the effects of processing conditions on the properties of the hierarchical composites, two key fabrication parameters were varied: nozzle temperature and electrospinning time. The nozzle temperature was investigated at three levels (210 °C, 220 °C, and 230 °C) to assess its influence on the interface bonding between the PLA matrix and the PAN nanofibers, as well as on the overall mechanical performance. The electrospinning time was varied (5 min, 10 min, and 15 min per layer) to control the amount of nanofiber reinforcement incorporated into each layer, allowing optimization of the nanofiber content for improved mechanical and thermal properties. All other processing parameters were kept constant across experiments to isolate the effects of these two variables.

### 2.3. Characterization

#### 2.3.1. Physical Properties

The mass and thickness of the fabricated specimens were measured to characterize their physical properties and to calculate the dimensional consistency under different processing conditions. Mass measurements were performed using a high-precision analytical balance (KERN ABT 5NM, KERN & Sohn GmbH, Balingen, Germany) with a maximum capacity of 100 g and a readability of 0.01 mg (0.00001 g), ensuring accurate and reliable quantification of the mass of each sample. The balance was calibrated according to the manufacturer’s specifications (serial number: WB22G0101).

Thickness measurements were performed using a digital micrometer (MDC-25PX, Mitutoyo Corporation, Kawasaki, Japan, serial number: 71912410) with a measurement range of 0 to 25 mm and a resolution of 0.001 mm. Multiple measurements were taken at different locations on each specimen to account for possible thickness variations, and the average value was calculated for stress and strain calculations in subsequent mechanical tests. These physical property measurements provided data to normalize the mechanical test results and assess the consistency of the fabrication process.

#### 2.3.2. Tensile Test

**Figure 2:**
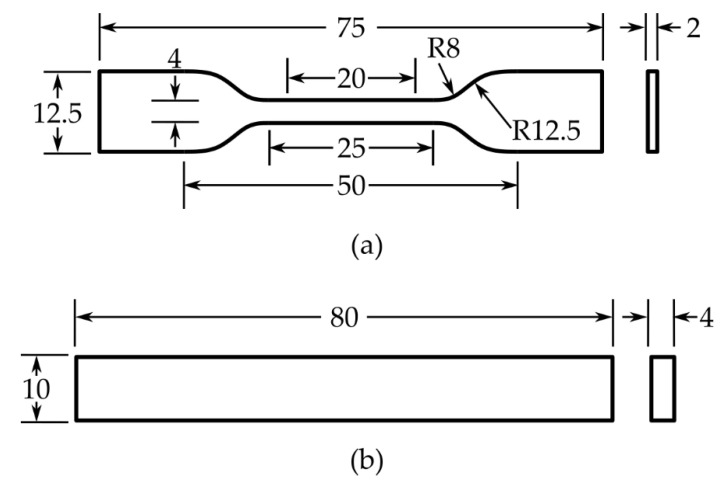
Geometries and dimensions (in mm) of specimens for mechanical testing: (**a**) tensile test according to the ISO 527-2 Type 5A standard, and (**b**) three-point bending test according to the ISO 178 standard.

Tensile testing was performed according to the ISO 527-2 Type 5A standard [49] to evaluate the mechanical properties of the fabricated hierarchical composite specimens. Dogbone-shaped tensile specimens were fabricated with dimensions as shown in Figure 2a: gauge length of 20 mm, gauge width of 4 mm, overall length of 75 mm, and thickness of approximately 2 mm. Before testing, the specimens were conditioned at 23 ± 1 °C and 40 ± 5% relative humidity for at least 24 h to ensure equilibrium moisture content and consistent test conditions.

Tensile tests were performed using a Mecmesin Multi-Test 2.5-i universal testing machine (PPT Group UK Ltd., West Sussex, UK) equipped with a 2500 N load cell. A Model 3442-020M-125M-ST miniature axial extensometer with a gauge length of 20 mm (Epsilon Technology Corp., Jackson, WY, USA) was attached to the gauge section of each specimen to accurately measure strain during testing. The crosshead speed was set to 1 mm/min in accordance with ISO 527-2. At least five specimens were tested for each condition to ensure statistical reliability.

The ultimate tensile strength (*σ_Tmax_*), Young’s modulus (*E_T_*), and tensile strain at break (*ε_T_*) were calculated from the stress–strain curves. Tensile stress was calculated as shown in Equation (1) [50,51]:

(1) $$\sigma_{T}=\;\frac{F}{A}$$

where *F* is the applied force (N) and *A* is the cross-sectional area of the gauge section (mm^2^). Young’s modulus was determined from the slope of the linear portion of the stress–strain curve, as shown in Equation (2):

(2) $$E_{T}=\;\frac{∆\sigma }{∆\varepsilon }$$

where Δ*σ* is the change in stress and Δ*ε* is the corresponding change in strain in the elastic region (between 0.05% and 0.2% strain). The tensile strain at break was calculated as shown in Equation (3):

(3) $$\varepsilon_{T}=\;\frac{∆L}{L_{0}}\;\times 100\%$$

where Δ*L* is the extension at the break and *L_0_* is the original gauge length (20 mm).

#### 2.3.3. Three-Point Bending Test

Three-point bending (3PB) tests were performed according to the ISO 178 standard [52] to evaluate the flexural properties of the composite specimens. Rectangular specimens with dimensions of 80 mm × 10 mm × 4 mm (length × width × thickness) were fabricated as illustrated in Figure 2b. Similar to the tensile specimens, all specimens were conditioned at 23 ± 1 °C and 40 ± 5% relative humidity for at least 24 h prior to testing.

The 3PB tests were carried out using a Mecmesin Multi-Test 2.5-i compression tester (PPT Group UK Ltd., West Sussex, UK) equipped with a three-point bending fixture and a 2500 N load cell. The test setup consisted of two support anvils with a span length (L) of 64 mm and a loading anvil with a diameter of 3 mm in the center. The loading anvil was positioned at the midpoint between the two support anvils, and the test was performed at a crosshead speed of 2 mm/min. At least five specimens were tested for each condition.

Flexural strength (*σ_Fmax_*), flexural modulus (*E_F_*), and flexural strain at failure (*ε_F_*) were calculated from the load–deflection curves. The flexural stress was calculated following Equation (4) [50,51]:

(4) $$\sigma_{F}=\;\frac{3FL}{2bh^{2}}$$

where *F* is the applied load (N), *L* is the support span (64 mm), *b* is the specimen width (10 mm), and *h* is the specimen thickness (4 mm). The flexural modulus was determined from the slope of the initial linear portion of the load-deflection curve, as shown in Equation (5):

(5) $$E_{F}=\;\frac{L^{3}m}{4bh^{3}}$$

where *m* is the slope of the initial linear portion of the load–deflection curve (N/mm). The flexural strain was calculated as stated in Equation (6):

(6) $$\varepsilon_{F}=\frac{6sh}{L^{2}}\times 100\%$$

where *s* is the deflection in the center of the specimen (mm).

#### 2.3.4. Thermogravimetric Analysis

Thermogravimetric analysis (TGA) was performed using a NETZSCH TG 209 F1 Libra^®^ thermomicrobalance (NETZSCH-Gerätebau GmbH, Selb, Germany) to evaluate the thermal stability and decomposition behavior of the hierarchical composite specimens. Small samples weighing approximately 6 ± 1 mg were carefully placed in aluminum oxide (Al_2_O_3_) crucibles. Thermal analysis was carried out by heating the samples from 20 °C to 800 °C at a constant heating rate of 10 °C/min under an inert nitrogen atmosphere with a flow rate of 30 mL/min to prevent oxidative degradation, following the protocol previously described by the authors [8]. The TGA curves provided information on the thermal decomposition temperatures, mass loss patterns, and thermal stability of both the PLA matrix and the PAN nanofiber reinforcement, as well as their interactions within the composite structure. The onset temperature of degradation (Tonset), maximum degradation temperature (Tmax), and residual mass at 800 °C were determined from the TGA and derivative thermogravimetric (DTG) curves.

#### 2.3.5. Differential Scanning Calorimetry

Differential scanning calorimetry (DSC) was performed according to the ASTM E1356 standard [53] using a DSC 214 Polyma differential scanning calorimeter (NETZSCH-Gerätebau GmbH, Selb, Germany) to characterize the thermal transitions of the hierarchical composites. Samples weighing approximately 6 ± 1 mg were placed carefully in standard aluminum crucibles with pierced lids and subjected to a two-cycle heating protocol to ensure accurate and reproducible thermal analysis.

The DSC measurement procedure consisted of the following steps: (1) initial heating from 0 °C to 200 °C at a rate of 10 °C/min to eliminate thermal history, moisture, and residual solvents; (2) isothermal holding at 200 °C for 1 min to ensure temperature equilibration; (3) cooling from 200 °C to 0 °C at a rate of 10 °C/min; (4) isothermal holding at 0 °C for 1 min; and (5) second heating from 0 °C to 200 °C at 10 °C/min, as previously described by the authors [46]. All thermal cycles were conducted under a nitrogen atmosphere with a purge gas flow rate of 40 mL/min to maintain an inert environment.

#### 2.3.6. Fracture Morphology Observation

The morphological characteristics of the hierarchical composite structures’ fracture surfaces were examined using a Hitachi TM3000 tabletop scanning electron microscope (SEM) (Hitachi High-Technologies Corporation, Tokyo, Japan), following the protocol previously described by the authors [8]. Before SEM imaging, the fractured specimens from the tensile and three-point bending tests were carefully mounted on aluminum stubs using conductive carbon tape. To enhance electrical conductivity and prevent charging effects during imaging, the samples were sputter-coated with gold (Au) to a thickness of 150 Å using an ion coater under vacuum conditions.

SEM imaging was performed at an accelerating voltage of 15 kV under a vacuum of 10^−2^ Torr, with magnifications ranging from 100× to 1500×, to observe both the overall fracture morphology and the fine details of the PLA-PAN interface, nanofiber distribution, and fiber–matrix adhesion. The fracture surface analysis provided information on the failure mechanisms, including fiber pulling, fiber breakage, and interfacial bond between the PLA matrix and PAN nanofibers.

Furthermore, the average diameter of the electrospun PAN nanofibers embedded within the composite structure was determined from SEM images using the ImageJ software (version 1.54h, National Institutes of Health, Bethesda, MD, USA) [54,55,56,57]. Measurements of fiber diameter were taken from 100 randomly selected nanofibers in three different SEM images for each sample condition to ensure statistical reliability. The diameter measurements were performed by drawing perpendicular lines across individual fibers, and the results are reported as mean ± standard deviation.

#### 2.3.7. Statistical Analysis

Statistical analysis was performed using Python (version 3.13, Python Software Foundation, Beaverton, OR, USA) with the SciPy library (version 1.16, SciPy) to evaluate the significance of differences between experimental conditions. ANOVA was performed to compare means across multiple groups, followed by Tukey’s honest significant difference (HSD) post hoc test for pairwise comparisons when significant differences were detected. The level of significance was established at α = 0.05, and *p*-values < 0.05 were considered statistically significant. All findings are reported as the mean ± standard deviation (SD) of at least five replicates (n ≥ 5) for each experimental condition to ensure statistical reliability.

## 3. Results and Discussion

### 3.1. Physical Properties of Specimens

The physical dimensions and mass of the fabricated specimens were measured to evaluate the reproducibility of the integrated FDM electrospinning process and to provide baseline data for calculations of mechanical properties. Table 3 presents the thickness and mass measurements for all experimental conditions.

Incorporation of PAN nanofibers resulted in statistically significant increases (*p* < 0.05) in both specimen thickness and mass compared to the neat PLA specimen (B1). Sample B2, manufactured with an electrospinning time of 10 min at an FDM nozzle temperature of 220 °C, showed thickness increases of 6.3% (tensile) and 6.3% (flexural) relative to B1, with corresponding mass increases of 6.5% and 6.6%, respectively. These dimensional changes reflected the addition of electrospun PAN nanofiber layers integrated into the structure of the PLA matrix.

Among the nanofiber-reinforced specimens (B2, T1, and T2), no statistically significant differences in thickness or mass were observed (*p* > 0.05), indicating that variations in nozzle temperature (210–230 °C) did not substantially affect the nanofiber deposition characteristics or the final specimen dimensions.

Electrospinning time had the most pronounced effect on physical properties. Sample E1 (5 min ES time) showed intermediate values between those of B1 and B2, with thickness and mass increases of 3.1% and 4.1%, respectively, compared to B1. Sample E2 (15 min ES time) exhibited the highest values, with thickness increases of 9.9% (tensile) and 9.9% (flexural) and corresponding mass increases of 15.8% and 15.6% compared to B1. These results indicate a linear relationship between the duration of electrospinning and the amount of nanofiber reinforcement incorporated.

The thickness increases observed were smaller than expected based solely on the addition of nanofiber mats, which can be attributed to the nanofiber–matrix integration mechanism during the layer-by-layer fabrication process. When the molten PLA filament (220–230 °C) was being deposited onto the electrospun PAN nanofiber mat, the low nozzle-to-bed distance (0.2 mm layer height) allowed the molten polymer to penetrate into the porous nanofiber network. This penetration allowed PLA to infiltrate and encapsulate the PAN nanofibers, creating an embedded reinforcement structure rather than discrete interleaved layers. For specimens with a shorter electrospinning time (E1: 5 min), the thinner nanofiber mats experienced nearly complete penetration of PLA, resulting in minimal thickness increase. On the contrary, with a longer electrospinning time (E2: 15 min), the thicker nanofiber mats showed partial penetration, with nanofibers present both within the PLA matrix and as interleaved layers, resulting in more substantial increases in thickness.

The observed physical property trends align with previous hybrid 3D printing–electrospinning studies. Khodabandeh et al. [36] reported similar dimensional increases when integrating electrospun PLLA nanofibers into PCL/HA microfibrous scaffolds, where the addition increased scaffold thickness due to the interleaved mat structure. He and Molnár [33] documented thickness variations of 5–10% when incorporating electrospun PLA nanofiber interleaves between FDM-printed PLA layers, comparable to the 6–10% range observed in specimens B2–E2.

### 3.2. Mechanical Characterization of Hierarchical Structures

#### 3.2.1. Tensile Test Results

**Figure 3:**
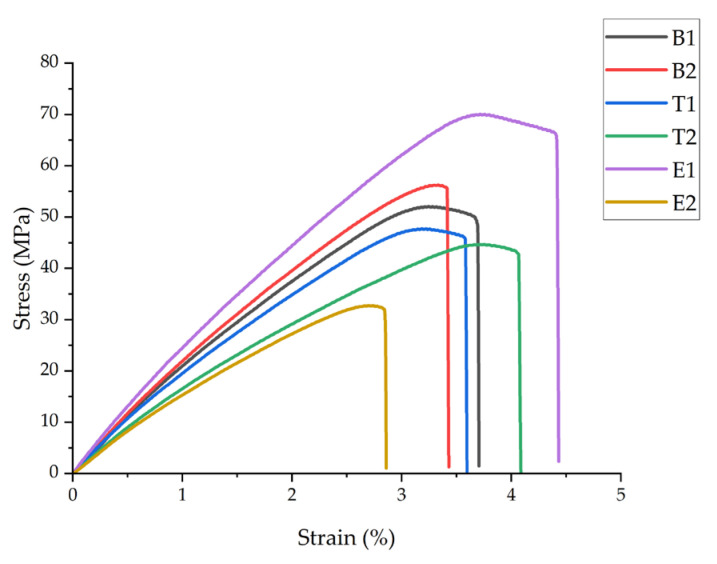
Representative tensile stress–strain curves for all sample groups, showing the stress–strain behavior of neat PLA (B1) and hierarchical structures with integrated PAN nanofibers (B2, T1, T2, E1, and E2).

Tensile testing was performed to evaluate the influence of PAN nanofiber reinforcement and processing parameters on the mechanical performance of the hierarchical structures. Figure 3 presents representative stress–strain curves, and Table 4 summarizes the ultimate tensile strength (*σ_Tmax_*), Young’s modulus (*E_T_*), and tensile strain at break (*ε_T_*).

Integration of PAN nanofibers at the optimal processing conditions (sample B2: FDM nozzle temperature of 220 °C, 10 min ES time) resulted in statistically significant improvements (*p* < 0.05) in tensile strength and modulus compared to the neat PLA (B1). Sample B2 exhibited an 11.5% increase in the ultimate tensile strength (58 ± 1.7 MPa vs. 52 ± 1.5 MPa) and a 14.3% increase in Young’s modulus (2.4 ± 0.06 GPa vs. 2.1 ± 0.05 GPa). However, the strain at break decreased slightly by 5.4% (3.5 ± 0.07% vs. 3.7 ± 0.08%), indicating a transition from ductile to more brittle behavior.

Temperature exhibited a pronounced effect on the mechanical properties. A lower printing temperature (T1: 210 °C) resulted in a 7.7% reduction in tensile strength compared to B1, while a higher temperature (T2: 230 °C) caused a more substantial decrease of 19.2%. Similarly, Young’s modulus decreased by 14.3% and 25.0% for T1 and T2, respectively. These temperatures deviated from the optimal 220 °C processing window. At 210 °C, an inadequate melt temperature likely reduced the mobility and penetration into the nanofiber network, resulting in poor interfacial bonding. At 230 °C, the low-viscosity molten PLA likely displaced and disrupted the nanofiber network during deposition, preventing proper fiber–matrix integration. The strain at break for T2 (4.2 ± 0.12%) increased by 13.5% compared to B1, suggesting thermal softening of the matrix despite reduced strength.

Electrospinning time demonstrated the most dramatic effect on mechanical performance. Sample E1 (5 min ES time) achieved the highest ultimate tensile strength (71 ± 2.1 MPa) and Young’s modulus (2.8 ± 0.08 GPa), representing improvements of 36.5% and 33.3%, respectively, compared to B1. The strain at break also increased by 21.6% to 4.5 ± 0.14%, indicating enhanced toughness. On the contrary, sample E2 (15 min ES time) exhibited the poorest mechanical properties, with 36.5% and 23.8% reductions in tensile strength and modulus, respectively, compared to B1. This inverse relationship between electrospinning time and mechanical properties can be attributed to the nanofiber mat thickness and the PLA infiltration efficiency. For E1, the thin nanofiber mat (5 min deposition) allowed for complete PLA penetration and encapsulation, creating optimal fiber–matrix interfacial bonding and load transfer. For E2, the thick nanofiber mat (15 min deposition) prevented complete PLA infiltration, resulting in poorly bonded interleaved layers that acted as stress concentrators and delamination sites during tensile loading. The improved mechanical performance of E1 can be further attributed to two mechanisms: First, the deposition of molten PLA (220 °C) onto PAN nanofibers created localized thermal annealing above 70 °C, which enhanced PAN nanofiber strength through increased molecular orientation and crystallinity [46,58,59]. Second, individual PAN nanofibers with diameters approximately 700 nm possessed an intrinsically high elastic modulus (2–4 GPa), depending on the processing parameters and molecular arrangement [60,61,62], providing effective reinforcement when properly integrated into the PLA matrix.

The mechanical properties of the neat PLA (B1) strongly align with prior studies [63,64,65] where neat PLA was tested. B1 exhibited a typical brittle fracture: a linear elastic response to ~2% strain, then yielding and failure at 3.7%. Nanofiber integration modified this—E1 maintained a high strength while increasing strain to 4.5% through load transfer and crack deflection mechanisms. The mechanical reinforcement mechanisms observed in this study align with previous research on hybrid manufacturing. Khodabandeh et al. [36] reported similar findings in PCL/HA scaffolds reinforced with aligned PLLA nanofibers, achieving a 268% increase in elastic modulus (from 6.4 ± 0.9 MPa to 17.16 ± 1.5 MPa) and a 130% increase in ultimate tensile strength (from 1.01 ± 0.5 MPa to 1.31 ± 0.07 MPa) compared to microfibrous scaffolds not reinforced by nanofibers. The lower percentage gain here (36.5% vs. 268%) reflects system differences: aligned fibers in porous scaffolds (neat 6.4 MPa) versus random fibers in dense PLA (neat 2.1 GPa), where a high initial stiffness limits relative improvement despite higher absolute values (71 MPa vs. 1.31 MPa). The nanofiber structure enabled efficient load transfer along the fiber axis, consistent with the improvements observed in sample E1. He and Molnár [33] documented increases of 16.5% in tensile strength and of 34.3% in Young’s modulus after integrating electrospun PLA nanofiber interleaves into PLA structures, which were comparable to the 11.5% and 14.3% increases observed in sample B2. Similarly, Lackner et al. [38] demonstrated that the control of fiber orientation in 3D-printed nanocellulose/alginate composites resulted in variations in tensile modulus ranging from 12.8 ± 0.6 MPa (perpendicular) to 23.4 ± 1.1 MPa (longitudinal), representing an increase of 83%, highlighting the critical role of fiber–matrix integration in hierarchical structures.

The observed reductions in mechanical properties at extended electrospinning times indicate that nanofiber content optimization is critical. The optimal reinforcement occurs when the thickness of the nanofiber mat reaches the molten PLA penetration depth, ensuring complete fiber wetting and interfacial bonding rather than discrete layer formation.

#### 3.2.2. 3PB Test Results

**Figure 4:**
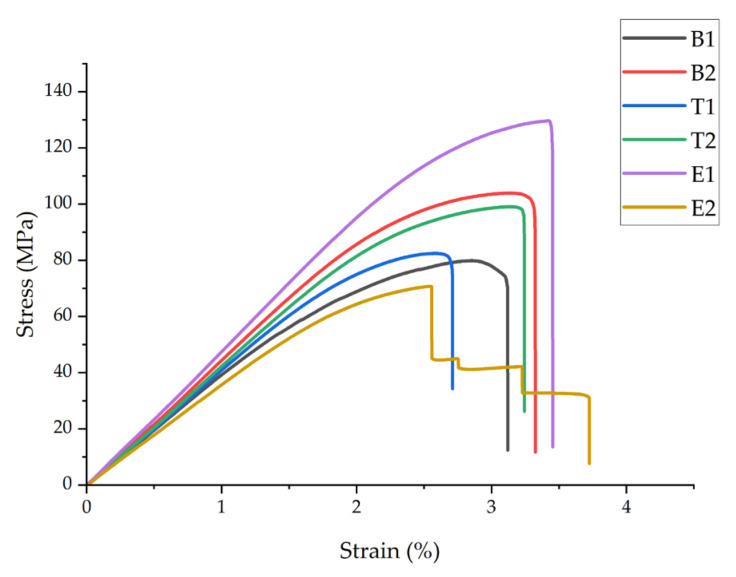
Representative flexural stress–strain curves for all sample groups, showing the stress–strain behavior under three-point bending for neat PLA (B1) and hierarchical structures with integrated PAN nanofibers (B2, T1, T2, E1, and E2).

Three-point bending (3PB) testing was performed to assess the flexural properties of the hierarchical structures under bending loads. Figure 4 shows representative stress–strain curves, and Table 5 summarizes the flexural strength (*σ_Fmax_*), flexural modulus (*E_F_*), and flexural deformation at failure (*ε_F_*) for all conditions.

The flexural properties exhibited trends similar to tensile behavior but with more pronounced improvements. Sample B2 demonstrated a 32.9% increase in flexural strength (105 ± 6.3 MPa vs. 79 ± 4.7 MPa) and a 14.3% increase in flexural modulus (4.0 ± 0.24 GPa vs. 3.5 ± 0.21 GPa) compared to B1, which were significantly higher than the tensile improvements observed (11.5% and 14.3%). The flexural strain at failure increased by 17.2% (3.4 ± 0.3% vs. 2.9 ± 0.2%), indicating a higher deformation capacity under bending loads. Statistical analysis confirmed significant differences (*p* < 0.05) between B1 and B2.

The effects on flexural properties differed from the effects on tensile behavior. Sample T1 (210 °C) showed only a 3.8% improvement in flexural strength relative to B1, while T2 (230 °C) achieved a 21.5% increase. The flexural modulus increased by 5.7% and 11.4% for T1 and T2, respectively. This contrasts with the reduced tensile properties at 230 °C. The difference arises from asymmetric stress distribution in bending: nanofiber displacement at 230 °C creates weak points in tension but has less impact on compression zones, where partial infiltration still provides reinforcement. Since flexural failure initiates at the outer surface under maximum stress, the compression-side reinforcement contributes to improved flexural strength despite poor fiber–matrix bonding in tension zones.

Electrospinning time again showed the strongest influence. Sample E1 exhibited the highest flexural strength (128 ± 7.7 MPa) and modulus (4.20 ± 0.25 GPa), representing 62.0% and 20.0% improvements, respectively, compared to B1. Sample E2 showed 13.9% and 5.7% reductions in flexural strength and modulus, respectively, compared to B1. The improved performance of E1 under bending relative to tension (increase of 36.5% vs. 62.0% in strength) indicates that optimal nanofiber integration provides greater reinforcement efficiency in flexural loading modes.

The flexural-to-tensile strength ratios provide insight into the behavior of the hierarchical structures. For B1, the ratio was 1.52, which was typical for brittle thermoplastics. For B2, it increased to 1.80, while E1 achieved a ratio of 1.81. These elevated ratios indicate that nanofiber integration improves bending resistance more effectively than uniaxial tension resistance, probably because of the occurrence of crack deflection and energy dissipation mechanisms in the layered structure during bending.

Comparison of the study results with the literature reveals competitive flexural performance. Lackner et al. [38] reported flexural modulus values ranging from 12.8 to 23.4 MPa for nanocellulose/alginate composites, depending on the fiber orientation, which were substantially higher than the values obtained in the present study due to the use of different materials and testing conditions (wet versus dry state). Khodabandeh et al. [36] documented significant improvements in mechanical properties through integration into tissue engineering scaffolds, although their focus was primarily on compressive and tensile testing. The present results demonstrate that layer-by-layer integration of electrospun nanofibers during FDM printing effectively enhances flexural properties when the processing parameters are optimized.

The consistently improved performance of E1 in both tensile and flexural tests confirms that a moderate nanofiber content (5 min ES time) with complete PLA infiltration produces optimal mechanical properties. This represents a critical design parameter for hierarchical structure fabrication, balancing reinforcement content with interfacial bond quality.

### 3.3. Thermal Characterization of Hierarchical Structures

#### 3.3.1. TGA Results

**Figure 5:**
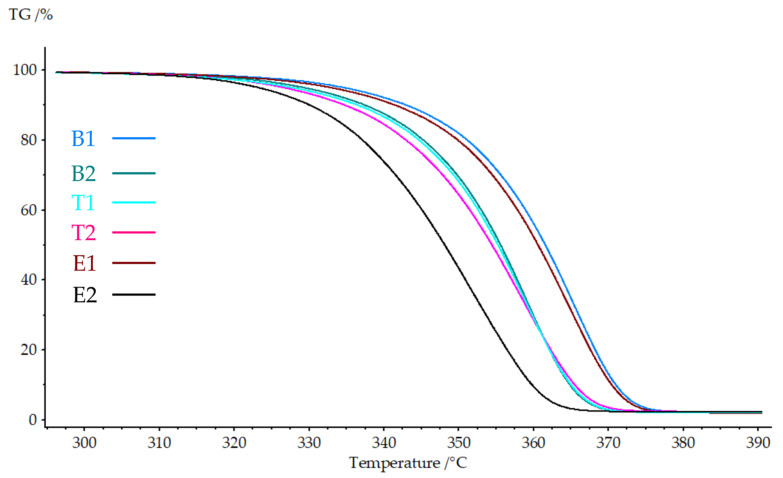
TGA curves showing the degradation region (300–390 °C) for all sample groups.

A thermogravimetric analysis was performed to evaluate the thermal stability and degradation behavior of the hierarchical structures. Figure 5 presents the TGA curves, while Table 6 summarizes the thermal degradation parameters.

All nanofiber-reinforced samples exhibited single-stage degradation typical of PLA-based materials, with complete decomposition occurring between 300 and 400 °C. The incorporation of PAN nanofibers resulted in decreased thermal stability relative to the neat PLA. Sample B2 showed reductions of 5.3 °C for Tonset, 5.5 °C for T5%, and 6.2 °C for Tmax relative to B1, indicating earlier onset of thermal degradation with nanofiber integration.

The effects on degradation behavior were minimal. Samples T1 and T2 exhibited a Tonset value of 345.9 °C and 343.7 °C, respectively, representing a reduction of 5.9 °C and 8.1 °C compared to B1. These small differences suggest that the printing temperature (210–230 °C) did not significantly alter the thermal decomposition characteristics of the hierarchical structures.

Electrospinning time demonstrated the most pronounced effect on thermal stability. Sample E1 maintained relatively high thermal stability, with a Tonset of 350.5 °C (only 1.3 °C lower than B1), while E2 showed substantial degradation, with a Tonset of 336.6 °C (15.2 °C reduction). The T5% and Tmax followed similar trends, with E2 exhibiting the lowest values across all parameters. This behavior correlates with nanofiber content: higher nanofiber loading (E2: 15 min) introduces more PAN material, resulting in lower thermal stability than PLA under a nitrogen atmosphere.

The reduced thermal stability of nanofiber-containing samples reflects the intrinsic thermal properties of PAN, which exhibits lower thermal stability than PLA under a nitrogen atmosphere. The increased interfacial area in the hierarchical structures also creates more sites for thermal decomposition initiation. The inverse relationship between nanofiber content and thermal stability is consistent with typical composite behavior, where reinforcement materials with lower decomposition temperatures reduce overall system stability.

These findings are consistent with those reported for polymer composites in the literature. Kara et al. (2023) [34] reported that PLLA fibers produced via melt blowing showed a glass transition temperature that was 4 °C higher and crystallinity that was 6% higher than the feedstock filament, demonstrating that processing-induced molecular orientation can influence thermal properties. However, the present study found that excessive nanofiber content (E2) overwhelms any potential thermal improvements from individual fiber properties, resulting in a net degradation of thermal stability.

#### 3.3.2. DSC Results

**Figure 6:**
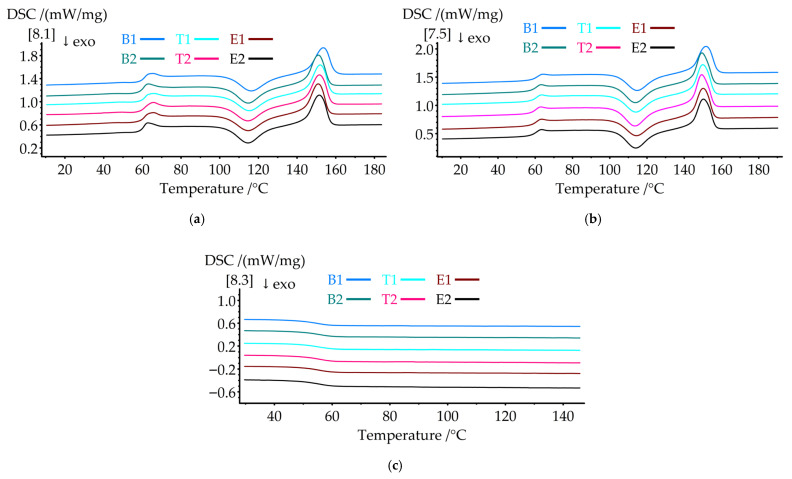
DSC thermograms showing (**a**) the first heating cycle with cold crystallization and melting peaks, (**b**) the second heating cycle after controlled cooling, and (**c**) the cooling cycle, illustrating the crystallization behavior for all specimen groups.

Differential scanning calorimetry was performed to characterize the thermal transitions and crystallization behavior of the hierarchical structures. Figure 6 presents the DSC thermograms of the (a) first heating, (b) second heating, and (c) cooling cycles. Table 7 summarizes the thermal transition parameters.

The glass transition temperature (Tg) decreased systematically with the integration of PAN nanofibers. Sample B2 showed a Tg of 59.3 °C, representing a 1.2 °C reduction compared to B1 (60.5 °C). All nanofiber-containing samples exhibited similar Tg values (59.2–59.5 °C), indicating that the processing parameters had minimal effect on molecular mobility. The decrease in Tg suggests that PAN nanofibers act as heterogeneous nucleation sites, disrupting PLA chain packing and reducing segmental mobility constraints. However, this 1.2 °C Tg reduction had minimal effect on the room-temperature (23 °C) mechanical properties, as the material remained well within the glassy state. The mechanical improvements observed for B2 resulted primarily from nanofiber reinforcement and load transfer mechanisms rather than changes in matrix glass transition behavior.

Although PAN has a higher Tg (85–95 °C) [46,66], its minor weight fraction in the composite (estimated <5 wt% for B2) was insufficient to shift the composite Tg upward. Additionally, the PAN Tg range coincides with the PLA cold crystallization temperature region (113–116 °C), where the strong crystallization exotherm masks any subtle PAN glass transition signal in the DSC curve. The observed Tg reduction reflects the plasticizing effect at the PLA-PAN interfaces, where disrupted PLA chain packing reduces segmental mobility constraints. The presence of a non-melting PAN phase throughout the structure also contributes to the altered molecular dynamics in the PLA matrix.

The cold crystallization temperatures (Tcc) shifted to lower values with nanofiber addition. In the first heating cycle, B2 exhibited a Tcc1 of 114.7 °C, which was 1.6 °C lower than that of B1 (116.3 °C). This trend persisted in the second heating cycle, where Tcc2 decreased from 114.7 °C (B1) to 113.8 °C (B2). The lower cold crystallization temperatures indicate enhanced crystallization kinetics, as PAN nanofibers provide nucleation sites that facilitate crystal growth at lower thermal activation energies. The temperature variations (T1, T2) and electrospinning time (E1, E2) had minimal influence on the Tcc values, with the values for all samples clustering around 114.5–114.7 °C (first heating) and 113.8–114.0 °C (second heating).

Cold crystallization enthalpy (ΔHcc) increased significantly with nanofiber content. Sample B2 showed ΔHcc1 of 20.7 J/g compared to 18.51 J/g for B1, representing an increase of 11.8%. Sample E2 exhibited the highest ΔHcc1 (21.3 J/g) and ΔHcc2 (24.8 J/g), indicating a greater amount of amorphous material being crystallized during heating. This behavior reflects incomplete crystallization during processing, indicating that nanofiber integration disrupts crystal formation.

Melting temperatures (Tm) decreased with nanofiber integration. The first heating reduced Tm1 from 153.5 °C (B1) to 150.9–151.5 °C for the nanofiber-containing samples. During the second heating cycle, the Tm2 values ranged from 149.5 to 150.0 °C for the hierarchical structures compared to 151.8 °C for B1. The reduction in Tm indicates the formation of less perfect, smaller crystallites in the presence of PAN nanofibers, which melt at lower temperatures because of surface energy effects. Melting enthalpy (ΔHm) remained relatively constant across all samples, with the values ranging from 25.79 to 26.9 J/g during the first heating cycle and from 24.3 to 25.0 J/g during the second heating cycle.

These results align with previous DSC studies on polymer composites. Kara et al. [34] reported that PLLA fibers showed a glass transition temperature that was 4 °C higher and crystallinity that was 6% higher than the feedstock filament due to shear-induced crystallization during processing. The present findings demonstrate opposite behavior: the presence of PAN nanofibers within the PLA matrix reduced the Tg and altered the crystallization kinetics, likely due to interfacial effects and disrupted chain packing rather than enhanced molecular orientation. This highlights that the thermal behavior of hierarchical structures is critically dependent on the specific integration mechanism and the interfacial interactions between components.

### 3.4. Fracture Morphology

**Figure 7:**
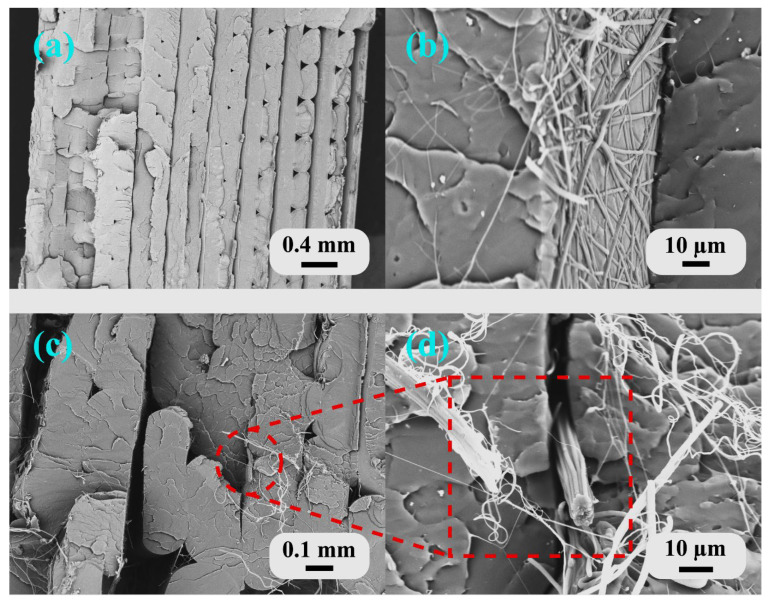
SEM images of tensile specimen fracture surfaces: (**a**) B1—neat PLA showing brittle fracture with smooth layer interfaces; (**b**) B2—nanofiber-reinforced structure showing fiber–matrix integration; and (**c**,**d**) T2—high temperature processing leading to nanofiber bundling between extrusion lines. Loading direction for all samples is perpendicular to the fracture surface shown. Different magnifications used to show overall fracture morphology and fiber–matrix interface details. Red dotted lines indicate magnified regions shown in adjacent panels.

Fracture surfaces were imaged using scanning electron microscopy to examine and elucidate the failure mechanisms of the hierarchical structures. Figure 7 shows the fracture morphology of the tensile specimens, revealing the interfacial characteristics between the PLA matrix and the PAN nanofibers under different processing conditions.

Sample B1 (neat PLA) exhibited the typical brittle fracture characteristics of FDM-printed thermoplastics (Figure 7a). The fracture surface showed distinct layer boundaries with relatively smooth surfaces, indicating weak interlayer bonding and minimal plastic deformation. The visible layer lines confirmed that crack propagation occurred preferentially along the interfaces between deposited filaments, a common failure mode in FDM-printed structures where interlayer adhesion is the weakest link. Similar layer-wise fracture patterns were observed in FDM-printed PLA structures in the study by Liesenfeld, J. et al. (2024) [67], confirming that interfacial failure dominates in unmodified 3D-printed structures.

Sample B2 (FDM nozzle temperature of 220 °C, 10 min ES) demonstrated successful integration of the nanofibers, with evidence of fiber–matrix bonding (Figure 7b). The fracture surface showed rougher topography compared to B1, with embedded nanofibers visible throughout the PLA matrix. The electrospun PAN nanofibers had an average diameter of 700 ± 85 nm. Diameter measurements were performed on delaminated and fractured samples where clear visibility of individual fibers was possible. Given constant electrospinning parameters across all experiments, the nanofiber diameter was considered uniform for all samples. These nanofibers appeared to be well distributed and partially encapsulated by the polymer, suggesting adequate melt infiltration during layer-by-layer deposition. The embedded nanofibers with adequate fiber–matrix bonding enabled effective load transfer and stress distribution throughout the structure, resulting in an 11.5% improvement in tensile strength observed for B2, as the integrated nanofibers provided reinforcement and altered the crack propagation path.

Sample T2 (FDM nozzle temperature of 230 °C, 10 min ES) revealed a different failure mechanism related to the excessive processing temperature (Figure 7c,d). The fracture surface showed significant nanofiber bundling and agglomeration between the extrusion lines. At 230 °C, the low-viscosity molten PLA dragged and displaced the electrospun nanofibers during deposition, creating fiber-rich bundles in the gaps between adjacent extrusion beads rather than uniform infiltration. These fiber bundles acted as stress concentrators and weak points, which explained the 19.2% reduction in tensile strength compared to B1. The disrupted nanofiber network was unable to effectively transfer loads, and the bundled regions likely acted as the initiation sites for premature failure.

**Figure 8:**
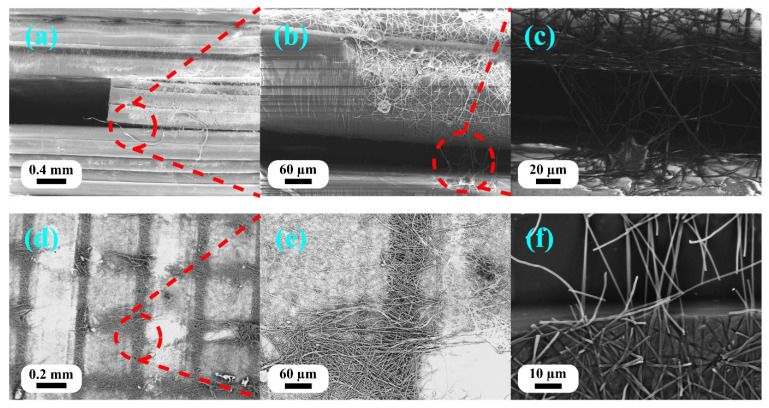
SEM images of three-point bending specimen fracture surfaces for sample E2 showing excessive nanofiber content effects: (**a**–**c**) poor fiber–matrix integration with nanofiber bundles remaining between 3D-printed layers (loading from top); (**d**,**e**) delaminated interface surfaces showing differential infiltration—partial fiber encapsulation beneath extrusion lines and uninfiltrated fiber mats in gaps (loading direction normal to image); and (**f**) delaminated PLA surface showing nanofiber impressions and fiber breakage, indicating weak fiber–matrix bonding (loading direction normal to image). Different magnifications used to illustrate delamination at macro-scale and fiber infiltration at micro-scale. Red dotted lines indicate magnified regions shown in adjacent panels.

Figure 8 shows the fracture morphology of the three-point bending specimens, demonstrating the delamination behavior and nanofiber–matrix interaction in structures with excessive nanofiber content.

Sample E2 of the three-point bending tests (FDM nozzle temperature of 220 °C, 15 min ES) exhibited severe delamination in the nanofiber-rich interlayers (Figure 8a–c). Multiple features of the fracture surface confirmed inadequate integration: (1) clear separation of distinct layers with the nanofiber mats remaining intact between PLA layers, (2) the presence of pulled-out nanofibers bridging delaminated surfaces, and (3) visible nanofiber impressions on the PLA surface, indicating contact without bonding (Figure 8f). The internal delaminated surface revealed differential infiltration patterns (Figure 8d,e): the nanofibers directly beneath the extrusion lines showed partial penetration and encapsulation, while the nanofibers in the gaps between the extrusion lines remained uninfiltrated and formed discrete mats. This non-uniform infiltration resulted from the thickness of the nanofiber mats (15 min deposition) exceeding the penetration depth of molten PLA. The poorly bonded interleaved layers acted as preexisting flaws that propagated rapidly under load, explaining the dramatic 36.5% reduction in tensile strength and 23.8% reduction in modulus compared to the neat PLA.

Fracture morphology observations elucidated the trends in mechanical properties. Sample E1 (5 min ES), although lacking direct SEM evidence, achieved improved mechanical properties (71 MPa in tensile strength, representing an improvement of 36.5%) because the thin nanofiber mat allowed complete PLA infiltration throughout its thickness. This created a true nanofiber-reinforced composite rather than a laminate structure. Balancing reinforcement benefits with interfacial bonding quality avoided both the insufficient reinforcement observed in B2 and the delamination issues seen in E2.

These findings are consistent with the hybrid manufacturing literature. Khodabandeh et al. [36] reported similar interfacial challenges in PCL/HA scaffolds with PLLA nanofibers, wherein the scaffold architecture and fiber distribution critically affected mechanical integration. He and Molnár [33] documented that the thickness of the nanofiber interleaves must be optimized to ensure proper bonding while maintaining the benefits of reinforcement. The present results demonstrate that hierarchical structure performance depends not only on nanofiber content but also on complete fiber–matrix infiltration by controlling the nanofiber mat thickness and processing temperature.

## 4. Conclusions

This study successfully demonstrated a novel layer-by-layer integration approach for fabricating hierarchical composite structures by combining 3D printing with in situ electrospinning of PAN nanofibers onto PLA matrices. The integrated system enables continuous, automated production of nanofiber-reinforced structures without sequential processing steps, addressing key limitations of existing hybrid manufacturing methods.

The electrospinning time emerged as the critical parameter controlling mechanical performance through its influence on nanofiber mat thickness and PLA infiltration. Sample E1 (5 min ES) achieved optimal properties, attaining 71 MPa in tensile strength (36.5% increase), 2.8 GPa in Young’s modulus (33.3% increase), 128 MPa in flexural strength (62.0% increase), and 4.20 GPa in flexural modulus (20.0% increase) compared to the neat PLA. This improved performance resulted from the complete penetration of molten PLA into the thin nanofiber mat, creating true fiber–matrix integration. On the contrary, excessive nanofiber content (E2: 15 min ES) caused delamination and a reduction of 36.5% in strength due to incomplete infiltration, resulting in poorly bonded interleaved layers. The nozzle temperature significantly affected the interface quality, with 210 °C producing insufficient melt flow and 230 °C causing nanofiber displacement and bundling, leading to degraded mechanical properties compared to the optimal temperature of 220 °C.

Thermal analysis revealed that the integration of PAN nanofibers decreased the glass transition temperature by 1.2 °C, shifted cold crystallization to lower temperatures (reduction of 1.6–1.8 °C), and reduced thermal degradation onset by 5.3–15.2 °C, depending on the nanofiber content. These changes reflected the interfacial disruption of PLA chain packing and the introduction of thermally less stable PAN domains. Fracture morphology confirmed the mechanisms: E1 showed embedded nanofibers with fiber–matrix bonding, while E2 exhibited clear delamination with differential infiltration patterns, wherein nanofibers beneath the extrusion lines were partially encapsulated but those between the extrusion lines remained uninfiltrated.

The research focused on the PLA/PAN material combination with random nanofiber orientation; therefore, the findings cannot be directly generalized to other polymer systems. Only compression and tensile/flexural mechanical tests were performed, and impact resistance, fatigue behavior, and long-term creep properties were not characterized.

The hierarchical structures manufactured in this study demonstrate potential for applications that require improved mechanical properties with lightweight design, including biomedical scaffolds, protective equipment, filtration membranes, and structural composites. Future work could explore aligned nanofiber deposition, alternative polymer combinations, optimization of layer-specific nanofiber content, and comprehensive characterization, including dynamic mechanical analysis and interface adhesion quantification.

## Figures and Tables

**Table 1:** Core parameters, which were kept constant across all experiments.

3DP/ES Parameters	Values
Printer chamber	Enclosure
Filament material	PLA
Filament diameter (mm)	1.75
Nozzle diameter (mm)	0.4
Layer thickness (mm)	0.2
Print speed (mm/s)	45
Infill density (%)	100
Raster angle	0/90
Top/bottom pattern	Rectilinear
Top/bottom Layers	1
Adhesive layer	Glue
Fan used	No
Bed type	Glass
Build orientation	flat
PAN solution (% w./w.)	10
ES flow rate (mL/h)	0.8
ES voltage (kV)	18
ES distance (mm)	15

**Table 2:** Experimental design of the parameters investigated.

Sample ID	Description	NF	Parameters	
			Nozzle Temperature (°C)	ES Time (min)
B1	Neat PLA	No	220	0
B2	PLA + Nanofibers (standard)	Yes	220	10
T1	PLA + Nanofibers (Low temperature)	Yes	210	10
T2	PLA + Nanofiber (High temperature)	Yes	230	10
E1	PLA + Nanofibers (Short ES time)	Yes	220	5
E2	PLA + Nanofibers (Long ES time)	Yes	220	15

**Table 3:** Physical properties related to tensile (T) and flexural (F) of the test specimens, showing thickness and mass variations under varying processing conditions.

	B1-T	B2-T	T1-T	T2-T	E1-T	E2-T
Thickness (mm)	1.91 ± 0.04	2.03 ± 0.05	2.04 ± 0.06	2.02 ± 0.05	1.97 ± 0.04	2.10 ± 0.06
Mass (g)	1.4234 ± 0.0389	1.5156 ± 0.0521	1.5089 ± 0.0487	1.5201 ± 0.0556	1.4823 ± 0.0412	1.6478 ± 0.0712
	**B1-F**	**B2-F**	**T1-F**	**T2-F**	**E1-F**	**E2-F**
Thickness (mm)	3.82 ± 0.09	4.06 ± 0.11	4.08 ± 0.10	4.05 ± 0.12	3.94 ± 0.08	4.20 ± 0.11
Mass (g)	3.3645 ± 0.1123	3.5876 ± 0.1456	3.5734 ± 0.1389	3.6023 ± 0.1501	3.5012 ± 0.1234	3.8912 ± 0.1678

**Table 4:** Summary of tensile test results.

Sample	Ultimate Tensile Strength, *σ_Tmax_* (MPa)	Young’s Modulus, *E_T_* (GPa)	Tensile Strain at Break, *ε_T_* (%)
B1	52 ± 1.5	2.1 ± 0.05	3.7 ± 0.08
B2	58 ± 1.7	2.4 ± 0.06	3.5 ± 0.07
T1	48 ± 1.4	2.2 ± 0.07	3.3 ± 0.1
T2	42 ± 1.2	1.8 ± 0.05	4.2 ± 0.12
E1	71 ± 2.1	2.8 ± 0.08	4.5 ± 0.14
E2	33 ± 1	1.6 ± 0.04	2.5 ± 0.07

**Table 5:** Summary of the 3PB test results.

Sample	Flexural Strength, *σ_Fmax_* (MPa)	Flexural Modulus, *E_F_* (GPa)	Flexural Strain at Failure, *ε_F_* (%)
B1	79 ± 4.7	3.5 ± 0.21	2.9 ± 0.2
B2	105 ± 6.3	4.0 ± 0.24	3.4 ± 0.3
T1	82 ± 4.9	3.70 ± 0.22	2.6 ± 0.2
T2	96 ± 5.8	3.90 ± 0.23	3.2 ± 0.3
E1	128 ± 7.7	4.20 ± 0.25	3.5 ± 0.3
E2	68 ± 4.1	3.30 ± 0.20	2.4 ± 0.2

**Table 6:** Summary of the TGA results.

Samples	B1	B2	T1	T2	E1	E2
Initial degradation Temperature Tonset (°C)	351.8	346.5	345.9	343.7	350.5	336.6
5% weight loss temperature, T5% (°C)	336.1	330.6	329.4	328.0	334.3	324.5
Maximum degradation Temperature Tmax (°C)	365.6	359.4	358.9	358.5	364.9	351.8

**Table 7:** Summary of DSC results.

Samples	B1	B2	T1	T2	E1	E2
Glass Transition Temperature, Tg (°C)—2nd heating	60.5	59.3	59.2	59.4	59.5	59.5
Cold Crystallization Temperature, Tcc1 (°C)—1st heating	116.3	114.7	114.6	114.5	114.6	114.5
Cold Crystallization Temperature, Tcc2 (°C)—2nd heating	114.7	113.8	113.9	114.0	113.8	113.9
Cold Crystallization Enthalpy, ΔHcc1 (J/g)—1st heating	18.51	20.7	20.6	20.8	19.9	21.3
Cold Crystallization Enthalpy, ΔHcc2 (J/g)—2nd heating	22.09	23.9	23.1	24.0	23.1	24.8
Melting Temperature, Tm1 (°C)—1st heating	153.5	151.0	151.5	151.3	150.9	151.5
Melting Temperature, Tm2 (°C)—2nd heating	151.8	149.5	150.0	149.6	150.0	150.0
Melting Enthalpy ΔHm1 (J/g)—1st heating	25.79	26.16	26.0	26.8	26.6	26.9
Melting Enthalpy ΔHm2 (J/g)—2nd heating	24.3	24.9	24.7	25.0	24.9	25.0

## Data Availability

Data is contained within the article.
